# Epithelial Cell Cycle Behaviour in the Injured Kidney

**DOI:** 10.3390/ijms19072038

**Published:** 2018-07-13

**Authors:** Lies Moonen, Patrick C. D’Haese, Benjamin A. Vervaet

**Affiliations:** Laboratory of Pathophysiology, Department of Biomedical Sciences, University of Antwerp, 2000 Antwerp, Belgium; lies.moonen@uantwerpen.be (L.M.); patrick.dhaese@uantwerpen.be (P.C.D.)

**Keywords:** AKI, CKD, cell cycle, proximal epithelium cell, G1/S arrest, G2/M arrest

## Abstract

Acute kidney injury (AKI), commonly caused by ischemia-reperfusion injury, has far-reaching health consequences. Despite the significant regenerative capacity of proximal tubular epithelium cells (PTCs), repair frequently fails, leading to the development of chronic kidney disease (CKD). In the last decade, it has been repeatedly demonstrated that dysregulation of the cell cycle can cause injured kidneys to progress to CKD. More precisely, severe AKI causes PTCs to arrest in the G1/S or G2/M phase of the cell cycle, leading to maladaptive repair and a fibrotic outcome. The mechanisms causing these arrests are far from known. The arrest might, at least partially, be attributed to DNA damage since activation of the DNA-damage response pathway leads to cell cycle arrest. Alternatively, cytokine signalling via nuclear factor kappa beta (NF-κβ) and p38-mitogen-activated protein kinase (p38-MAPK) pathways, and reactive oxygen species (ROS) can play a role independent of DNA damage. In addition, only a handful of cell cycle regulators (e.g., p53, p21) have been thoroughly studied during renal repair. Still, why and how PTCs decide to arrest their cell cycle and how this arrest can efficiently be overcome remain open and challenging questions. In this review we will discuss the evidence for cell cycle involvement during AKI and development of CKD together with putative therapeutic approaches.

## 1. Introduction

Our kidneys play a crucial role in maintaining body homeostasis, i.e., they provide a balanced ionic composition, volume, pH and osmolality of our body fluids, thereby ensuring proper functioning of all our body cells. Hence, injured or diseased kidneys have far-reaching consequences for the health of an individual. Malfunction of the kidneys can occur either abruptly (termed acute kidney injury, AKI) or develop progressively (termed chronic kidney disease, CKD). According to the KDIGO guidelines, AKI can be characterized by several definitions: (i) an increase in serum creatinine (>0.3 mg/dL) within 48 h; (ii) an increase in serum creatinine to 1.5 times baseline values within seven days; or (iii) a urinary output <0.5 mL/kg/6 h [1]. AKI has different aetiologies which can be divided in three main groups: prerenal (e.g., decreased blood flow to the kidneys, ischemia-reperfusion injury), intra-renal (e.g., glomerulonephritis) and postrenal (e.g., ureter obstruction) [2]. Together with nephrotoxins, ischemia-reperfusion injury is the main cause of AKI. Despite increased understandings of AKI and its aetiologies, the incidence of AKI has currently reached 5–7% in hospitalized patients, and is rising [2].

CKD, on the other hand, is defined as abnormalities of kidney structure or function, present for >3 months, with implications for health [3]. CKD has a high global prevalence of 13.4% [4]. However, a recent epidemiological study in Morocco in which multiple independent serum samples were collected for creatinine measurement demonstrated that the prevalence of CKD is often overestimated [5]. CKD is not only a disease of the kidneys, it also affects other organ systems (especially the cardiovascular system and mineral/bone metabolism) as evidenced by an increased risk of cardiovascular disease (CVD) [3] and bone disorders [6]. Altogether, it is obvious that these complications are associated with higher morbidity and mortality and also have an important socio-economic impact [7]. Major risk factors for CKD include diabetes [8], hypertension [9], obesity [10] and older age [11]. Other important risk factors that are less frequent include toxic insult, glomerulonephritis, lupus and polycystic kidney disease [12]. CKD is histopathologically characterized by a progressive deposition of extracellular matrix (ECM) proteins (e.g., fibronectin and collagens), termed chronic fibrosis [13]. Mostly, this is accompanied by tubular atrophy and alterations in the renal vasculature [13,14].

Decades of research have made clear that proximal tubular epithelial cells (PTCs) play an important role in the histopathology of renal injury, both in AKI and CKD. After an acute ischemic or toxic insult, PTCs (particularly those of the proximal S3 segment) are most vulnerable and susceptible to injury [15]. Ischemia-reperfusion and drug-induced renal injury lead to increased production of reactive oxygen species (ROS) heavily contributing to PTC injury [16,17]. The sensitivity of the PTCs is due to (i) the high metabolic activity/demand of these cells; (ii) physiological hypoxia in the medullary region; and (iii) high exposure to intra-tubular toxins due to upstream water absorption [17,18]. Remarkably, PTCs have a strong inherent ability to regenerate after injury. This duality was nicely demonstrated by Grgic et al. in an animal model in which PTCs expressed the diphtheria toxin receptor and hence could be specifically targeted by this toxin [19]. A single administration resulted in PTC cell death and a vigorous inflammatory response, but epithelial repair was adaptive and injury was reversible. Upon triple administration, however, repair was maladaptive with sustained injury and interstitial fibrosis, which are hallmarks of CKD [19,20]. The biology of the PTC is thus an important determinant in renal injury and repair.

An important aspect herein is its proliferative behaviour. In vivo cell fate tracing studies have shown that repair of injured tubules does not involve specialized progenitors, but that proliferation of PTCs itself is the key of renal repair [21,22]. Thus, despite their sensitivity to injury, this cell type has a significant regenerative capacity and contributes to recovery of renal function by replacing deathly injured PTCs through proliferation [23]. Initially, PTCs become flattened and lose their polarity and brush border [24]. Heavily injured cells will undergo apoptosis or necrosis, whereas sublethally injured cells restore the tubular epithelium by rapidly re-entering the cell cycle [21,25]. Despite the great regenerative capacity of the kidney, an episode of AKI leaves its marks and repair is often incomplete, which can lead to the development of CDK. Studies of the last two decades have made clear that cell cycle behaviour plays a crucial role in determining the renal functional and histopathological outcome after AKI. In this review, we summarise our current molecular insights on (aberrant) cell cycle behaviour in AKI and CKD.

## 2. The Cell Cycle: A General Overview

**Figure 1:**
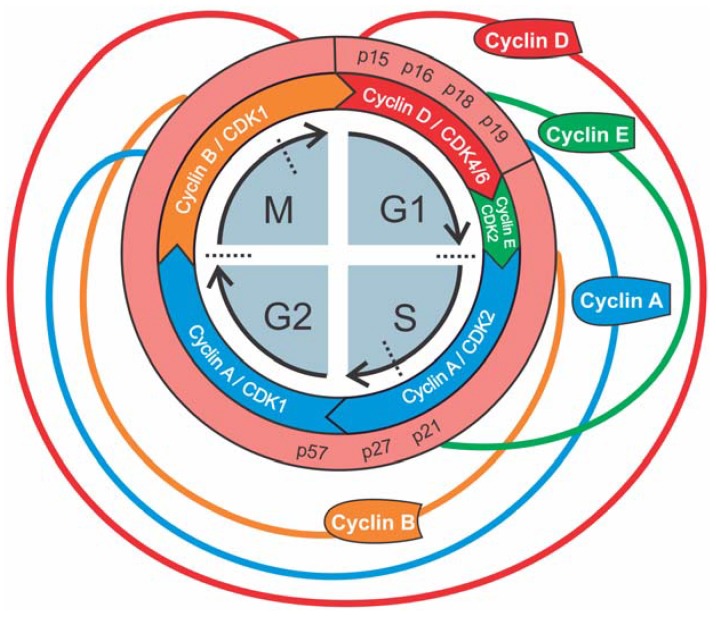
Expression pattern of different cyclin proteins and CKIs during various phases of the cell cycle. Dotted lines represent cell cycle checkpoints.

In eukaryotes, the cell cycle consists of four distinct phases (G1, S, G2, M), each with specific molecular characteristics (Figure 1) [18]. Cell division starts with the G1 phase. During this phase, cell growth takes place as well as synthesis of mRNA and proteins required for DNA duplication. The phenotypical status in which the cell resides before entering the G1 phase is often referred to as G0 phase. This is a resting stage during which the cell is in a quiescent non-proliferative state [18]. DNA replication is confined to a discrete synthesis or S phase. This is followed by the G2 phase, which is a period of rapid cell growth and protein synthesis during which the cell prepares itself for mitosis or M phase. During the M phase, genetic material and cellular components are precisely divided between two daughter cells. This phase is further subdivided in five stages: prophase, metaphase, anaphase, telophase and cytokinesis.

Progression through the cell cycle is mediated by three different classes of proteins: cyclins, cyclin-dependent kinases (CDKs) and cyclin-dependent kinase inhibitors (CKIs). CDKs are a family of serine/threonine protein kinases ranging from CDK1 to CDK13. Within this family, only CDK1, CDK2, CDK4 and CDK6 are involved in the cell cycle. At baseline, when CDKs are not coupled with their specific cyclins (A, B, D and E) they have very low levels of activity. Only when cyclin/CDK complexation occurs they become active and induce serine/threonine phosphorylation of their downstream targets to facilitate cell cycle progression. Although concentrations of CDKs remain more or less stable during proliferation, the sequential availability of cyclins guide the cell through each specific phase of the cell cycle (Figure 1). Cell cycle progression is negatively controlled by inhibitors of CDKs, i.e., CKIs. These inhibitors are divided in two main groups; INK4 and CDK2 interacting protein (CIP)/kinase inhibiting protein (KIP). INK4 proteins (inhibitors of CDK4; p15, p16, p18, p19) inhibit the CDK4/6-cyclin D complex. CIP/KIPs, such as p21, p27 and 57, exhibit a wider activity against other cyclins/CDKs. DNA damage or physiologic stress can lead to the induction of these CKIs and thus inhibit progression of the cell cycle.

To ensure the fidelity and integrity of the cell cycle process, progression from one phase to the next is controlled by a series of checkpoints, which were first described by Hartwell and Weinert in 1989 [26]. These checkpoints ensure that a cell can only progress through the different stages if it, and in particular its DNA, is in a suitable condition. If not, the cell cycle is halted, and repair mechanisms are activated. There are four main checkpoints: G1/S checkpoint, intra-S checkpoint, G2/M checkpoint and intra-M checkpoint. In the following paragraphs we will discuss our current understanding of aberrant proximal epithelial cell cycle behaviour for each cell cycle phase.

## 3. Cell Cycle Phases of Proximal Tubular Epithelial Cells in Health and Renal Injury

### 3.1. G0 Phase

Under normal conditions, the fraction of proliferating tubular epithelial cells in the kidney is below 1% [27] and balances the casual loss of tubular epithelial cells due to physiological cell death or spontaneous release from the basal membrane into the urine [28]. The remaining 99% cells are called “quiescent cells” and are resting in the G0 phase. However, for the proximal tubular epithelium, this is only partly true. Vogetseder et al. demonstrated that 20% of S1/S2 cells and 40% of S3 cells express cyclin D, which is a marker of the mid-to-late G1 phase [29]. Additionally, nearly all S3 cells were immunoreactive for p27, a CKI that blocks cell cycle progression and keeps cells in the G1 phase [30,31,32]. It is assumed that this physiological G1 arrest ensures that PTCs, after an ischemic or toxic insult, can initiate proliferation extremely rapid (i.e., proliferative burst) in order to replace the vast number of cells that have died by necrosis and apoptosis [15,33].

### 3.2. G1/S Checkpoint

The G1/S checkpoint evaluates sufficient cell size and the absence of DNA damage by multiple, complex and interdependent regulatory pathways. In order to ensure that each daughter cell is endowed with the correct amount of genetic and biosynthetic material, cells must grow about double their contents before division. To control cell size, it is thought that some product of translation, the so-called ‘translational sizer’, rises with cell size and stimulates progression through the cell cycle after a certain amount of protein has accumulated. Both cyclin D and cdc25, which is the most important phosphatase for cell cycle regulation, are proposed translational sizers.

**Figure 2:**
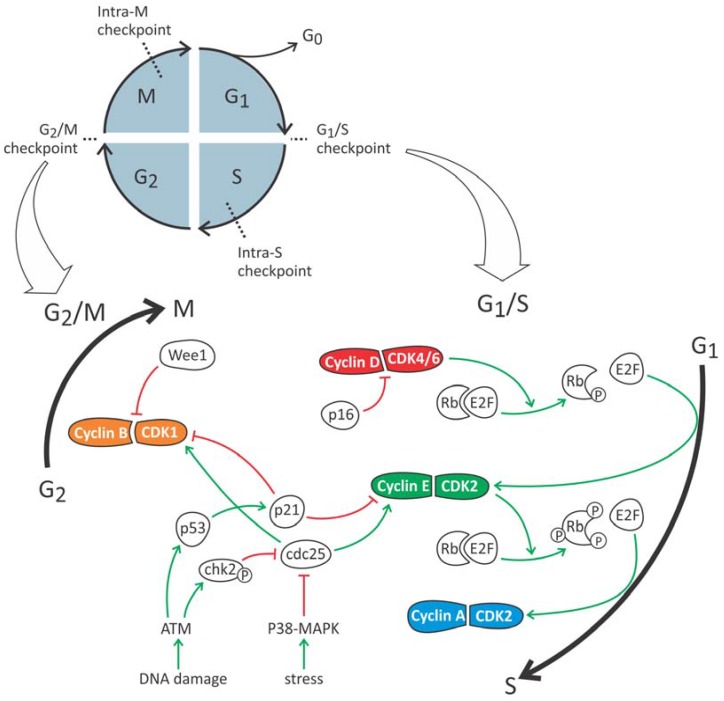
Pathways involved in cell cycle progression and arrest during G1/S and G2/M transition.

On the molecular level, the concentration of cyclin D, which determines the activity of CDK4 and CDK6, is the highest during the G1 phase (Figure 1). Activated CDK4/6 induces phosphorylation of the retinoblastoma protein (Rb) (Figure 2). When not phosphorylated, Rb inhibits the activity of E2F, which is a transcription factor for genes necessary for the transition to the S phase (such as cyclin E). Phosphorylation of Rb leads to decreased E2F inhibition, which in turn induces cyclin E production and initiation of the S phase. At the end of the G1 phase, cyclin D concentration drops as well as CDK4/6 activity. The newly formed cyclin E complexes with CDK2 and hyperphosphorylates Rb, leading to further increased E2F and cyclin E activity. This positive feedback loop drives the cells irreversibly to the S phase where cyclin A takes control and activates CDK2.

DNA damage has been confirmed in many AKI models, including ischemia, acute aristolochic acid toxic nephropathy (AAN) and unilateral ureteric obstruction (UUO) [34]. Upon DNA damage, ataxia telangiectasia mutated (ATM) and/or ataxia telangiectasia and Rad3-related protein (ATR), which belong to the phosphaditylinositol 3-kinase family, acts as sensors and phosphorylate several downstream targets including p53 and checkpoint kinase 2 (CHK2). CHK2 in turn phosphorylates cdc25, leading to its ubiquitination and degradation. Therefore, cdc25 can no longer activate the previously mentioned CDK2/cyclin E, and PTCs will remain in G1 phase. In order to maintain this arrest, an extra response is initiated. ATM/ATR also phosphorylates tumor suppressor p53, which leads to the production of p21, a CKI which inhibits CDK2/Cyclin E complexation by promoting degradation of cyclin E. In addition, another mechanism is activated involving p16. This CKI binds CDK4/6, blocking its interaction with cyclin D, thus keeping the cell from transitioning to the S phase.

Together with an increase in proliferation after AKI, there is a rapid induction of p21, a cell cycle inhibitor known to inhibit CDK2, thereby arresting PTCs in the G1 phase [35,36]. This appears contradictory as stressing the kidney leads to an increase in proliferation, whereas, at the same time, this stress induces a cell cycle inhibitor. A prolonged stay in G1, however, is not per se detrimental as it makes time for DNA repair, thereby avoiding propagation of genetic defects. In addition, it prevents cells from dividing if insufficient bio-energetic resources are available during stress [36].

Besides critical levels of DNA damage, high levels of reactive oxygen species (ROS) are considered a major cause of senescence induction [37,38]. ROS are molecules that contain at least one unpaired electron in their highest occupied atomic or molecular orbital. Therefore, these radicals are extremely reactive [39]. ROS can originate either from an exogeneous source (e.g., pollutants, ultraviolet radiation, tobacco smoke, ionising radiation) or from an endogenous source. The cellular produced ROS can be subdivided into two groups: actively produced ROS or production as a by-product of a relevant biological process (e.g., mitochondrial respiratory chain) [39]. The major source of actively produced ROS are nicotinamide adenine dinucleotide phosphate (NADPH) oxidases (NOX). Seven isoforms of ROS have been identified from which NOX4 is predominantly found in the kidney and is thus the major producer of renal ROS. NOX2 is predominantly found in phagocytotic cells [40]. For a long time, ROS were commonly thought to have only toxic effects, resulting in oxidation of various cell constituents such as DNA, lipids and proteins, eventually leading to cell damage and cell death [39]. However, ROS also play an important role in physiologic cell processes such as cell signalling as second messengers [41], mediating hormonal effects [42,43], oxygen sensing [44], regulation of ion channel activity, gene expression [45], adipocyte differentiation [46], reproduction [47], cell growth, senescence, apoptosis [48] and cell cycle progression. Depending on its amount, ROS can cause many cellular responses, such as transient growth arrest, permanent growth arrest, apoptosis and necrosis. Low levels of ROS have been implicated in causing an increase in cell cycle progression. In this latter case, ROS usually have an endogenous origin. ROS affect cell cycle progression mainly via ubiquitination/phosphorylation of CDKs and other cell cycle regulatory molecules such as cdc25 and CKIs. As mentioned above, cdc25 is the most important stimulator of cell cycle progression [49]. ROS can influence cdc25 activity by enhancing its phosphorylation, or alternatively by its inactivation via sulfonation of cysteine in the active site [50]. However, Yamaura et al. showed that inhibition of ROS production by NOX4 leads to hyperphosphorylation and inactivation of cdc25 in melanoma cells [51]. This proves that the effect of ROS is context dependent and may be related to local and/or temporal differences. Other studies also have demonstrated that oxidative stress induces phosphorylation and activation of MAPKs. ROS also affects expression of CKIs by ubiquitination and subsequent degradation, which are important mechanisms contributing to the irreversibility of the cell cycle. ROS decreases ubiquitination by inhibition of E1, E2 and proteasomes. In this way, ROS promotes progression from G1 to S phase by inhibition of cyclin A ubiquitination. Treatment with antioxidants on the other hand, prevents accumulation of cyclin A and thus results in G1 phase arrest. In addition, recent evidence shows that ROS are able to activate important growth factor receptors such as epidermal growth factor receptor (EGFR) and thereby promote cell cycle progression [52]. However, renal injury induced by ischemia-reperfusion leads to excessive production of ROS beyond the scavenging capacity of this organ, impairs antioxidant enzymes and causes cell damage by lipid peroxidation, DNA breakdown, and protein damage [53]. The ultimate effect of increased ROS on cell cycle progression is, however, difficult to predict due to the complex nature of ROS effects on different cellular processes. It depends on the complex nature of the molecular network that regulates cell cycle progression, the location of its production and the type of ROS. Rueckert and Mueller [39] were the first ones to observe the decreased proliferation in HeLa cells in response to oxygen-induced cytotoxicity. Since then, several studies have demonstrated that ROS can lead to G1 arrest. However, both increases cell proliferation and G1 phase growth arrest have been observed after oxidative stress [39].

### 3.3. Intra-S Checkpoint

During the S phase, a second checkpoint comes in to control DNA replication. This intra-S checkpoint involves the previously mentioned ATM/ATR machinery, which is activated upon DNA damage with activation of CHK2, cdc25 phosphorylation and CDK2 inhibition and ultimately blocks the recruitment of DNA polymerase α needed for replication [54]. Whether this checkpoint plays a role in AKI–CKD is currently not known.

### 3.4. G2/M Checkpoint

At the start of the G2 phase, the CDK1/cyclin A complex is most active, but currently not much is known about its role in G2-M transition. More important is the CDK1/cyclin B complex, the concentration of which rises in the late-G2 phase and is responsible for initiation of mitosis (Figure 1). This complex is also the ultimate target of pathways that mediate G2/M arrest controlled by the G2/M checkpoint. This checkpoint makes sure that cells are blocked from entering mitosis unless DNA replication/repair is complete and cell size is sufficient. Mediators involved in this arrest are the ATM/ATR pathway [55], stress-induced p38-MAPK pathway [56] and Wee1 (i.e., mitosis inhibitor protein kinase) [57] (Figure 2).

DNA damage leads to the activation of the ATM/ATR pathway, following CHK2 activation which phosphorylate p53 and cdc25, resulting in G2/M arrest by CDK1 inhibition [55,58,59,60]. In normal conditions, this arrest ensures time for DNA to repair before continuing to mitosis. The involvement of this pathway is demonstrated by studies in patients with Fanconi anemia-associated nuclease 1 (FAN1) mutations [61]. Cells exhibiting this mutation are more susceptible for DNA damage and genome instability and undergo G2/M arrest. This leads to the development of tubular atrophy and fibrosis in FAN1 patients [61]. Although it has well been established that DNA damage can activate ATM/ATR signalling, non-DNA-damaging conditions such as hypoxia and reoxygenation also stimulate this pathway [62].

G2/M arrest can also be induced by different stressful stimuli (e.g., hypoxia) via the p38-MAPK pathway. MAPK has been shown to phosphorylate cdc25, leading to its ubiquitination. As a result, cdc25 can no longer activate the CDK1/cyclin A complex, thereby inhibiting G2-M transition [63].

A third important mediator of G2/M arrest is Wee1 kinase which keeps CDK1 in an inactive state through phosphorylation of its tyrosine residue [64]. Re-activation of CDK1 occurs through dephosphorylation by cdc25 [65].

An important finding corroborating the involvement of G2/M arrest in renal injury was reported by Yang et al. in 2010 [34]. By investigating tubular epithelial cell cycle behaviour after renal injury, they noted a causal relationship between epithelial cell cycle arrest at the G2/M phase and a subsequent development of renal fibrosis due to maladaptive repair. Hereto, they characterized the cell cycle profile of PTCs after an acute insult in five experimental mouse models of AKI: moderate bilateral ischemia-reperfusion injury (IRI), severe bilateral IRI, unilateral IRI, AAN and UUO. These models reflect the three most common causes of AKI seen in humans: ischemia, toxic exposure and obstruction [66]. In all animal models, except moderate IRI, injury led to the development of severe fibrosis demonstrating the chronic fate of the kidney. In particular, they found that cell cycle arrested PTCs produce an increased amount of pro-fibrotic growth factors, such as transforming growth factor beta (*TGFβ*), Collagen 1 (*Col Ι*) and *CCN2*, formerly known as connective tissue growth factor (*CTGF*) [34,67,68,69]. This establishes a pro-fibrotic micro-environment by which resident fibroblasts become permanently activated [70], i.e., they persistently proliferate and excessively deposit ECM [15,71,72]. Thus, these tubular cells assume a so-called ‘senescent associated secretory phenotype’ (SASP). Moreover, pharmacologic intervention which increases the number of G2/M-arrested cells after injury worsens fibrotic outcome, whereas intervention which facilitate movement through G2/M are associated with less fibrosis [18,73,74]. This research of the last decade made clear that AKI is linked to the later development of CKD through PTC cell cycle events.

Occurrence of DNA damage has been confirmed in many AKI models, including ischemia, AAN and UUO [34]. This leads to the activation of the ATM/ATR signalling pathway and its downstream regulatory proteins p21 and p53, hence, affects cell cycle behaviour. p21 was one of the first proteins to be explored by Megyesi et al. [75,76,77]. Interestingly, this protein seems to exert a different effect during AKI and CKD progression. p21 seems to be protective during AKI, as assessed by a more pronounced renal dysfunction and mortality in p21^−/−^ mice [75,77], whereas during progression of CKD after 5/6th nephrectomy, p21^−/−^ mice developed less histopathological lesions [76]. p53, a regulator of p21 transcription, is also upregulated after AKI. Inhibition of p53, by knocking-out or blocking with pifithrin-α, leads to a reduction of renal lesions in AAN [78,79]. Aristolochic acid represses the degradation of p53 leading to increased expression of its downstream targets including p21. As p21 is a universal CDK inhibitor, it will inhibit CDK1/cyclin B complexation and thereby prevent G2/M transition [80].

Another important finding was reported by Borst et al. showing that c-Jun NH2-terminal kinase (JNK), an important mediator of the MAPK signalling pathway, remains activated in PTCs even weeks after severe ischemic injury [81]. This persistent activation was due to G2/M-arrested cells and resulted in upregulation of pro-fibrotic cytokines [34]. In addition, secretion of some pro-inflammatory cytokines such as IL-8 by arrested cells or neighbouring cells, promote G2/M cell cycle arrest. IL-8 binds a CXCR2 receptor leading to even more activation of the p83-MAPK pathway, which is responsible for cell senescence [82].

Finally, also ROS can cause a G2/M arrest. Elevation of ROS after injury was demonstrated to result in p53-dependent accumulation of p21, leading to G2/M cell cycle arrest [83,84].

### 3.5. Intra-M Checkpoint

During the M phase, a fourth and last checkpoint is involved, i.e., the intra-M or spindle checkpoint. Separation of sister chromatids during anaphase is an irreversible step. The spindle checkpoint examines whether all sister chromatids are correctly orientated and attached to the spindle microtubules. Separation is triggered by the anaphase-promoting complex (APC). Kinetochores that are not connected to spindle microtubules secrete a signal that inhibits APC, thereby preventing cell separation of chromatids [85]. As for the intra-S checkpoint, the involvement of this checkpoint in AKI and CKD remains unknown up till now.

## 4. Cell Cycle Intervention as Potential Innovative Strategy in the Treatment of Renal Injury

It is clear that cell cycle arrest has beneficial as well as deleterious effects on the development of AKI and CKD [34,36]. Therefore, therapeutic strategies which promote or prevent cell cycle arrest could be of great value in the treatment of renal diseases. However, they should be developed with great care since inappropriate interference with cell cycle events can easily lead to cell death and fibrosis or malignancy.

### 4.1. G1/S

Experiments with genetic mouse models and pharmacological inhibition have shown that deliberately increased expression of the cell cycle inhibitor p21, as well as decreased expression of CDK2, induces G1 arrest which ameliorates AKI by protecting against apoptosis of PTCs [75,77,86]. Unfortunately, all currently known CDK2 inhibitors are not specific as they also inhibit other CDKs (especially CDK7 and CDK9) which are involved in transcription, DNA-damage response (DDR) and DNA metabolism, leading to G2/M arrest and intra-S phase arrest, thereby promoting apoptosis and fibrosis [86,87].

Recently, two CDK4/6 inhibitors (Palbociclib and Ribociclib) were approved by the Food and Drug Administration (FDA) for the treatment of metastatic breast cancer [88]. Application of these inhibitors for the treatment of renal injury has shown their utility by inducing G1 cell cycle arrest in mice that underwent bilateral IRI [89,90]. DiRocco et al. demonstrated that this “pharmacological quiescence” induced by these CDK4/6 inhibitors protects PTCs from DNA damage and caspase activation [90]. It is important to note that CDK4/6 inhibition by Palbociclib and Ribociclib is only temporary and cells re-enter cell cycle 36–48 h after treatment [91]. This is an important asset, as sustained G1 arrest can lead to hypertrophy and fibrosis [92].

Given the beneficial as well as unfavourable pleiotropic effects of inducing G1 cell cycle arrest on the level of the cyclins and CKDs, it is important to note that alternative strategies aimed at interfering with downstream pathways are being investigated. Derynck et al. and Matsuura et al. have shown that extensive G1 arrest leads to increased expression of TGFβ through the TGFβ/Smad3 pathway and that it is responsible for the development of fibrosis following sustained G1 arrest [93,94]. Inhibition of this pathway with Smad7 has shown to protect against AKI by rescuing PTCs from G1 cell cycle arrest [95].

### 4.2. G2/M

The fact that G2/M arrest plays such an important role in the progression to CKD opens up new therapeutic perspectives. Targeting G2/M cell cycle arrest can be done by different approaches: (i) facilitation of movement through the G2/M checkpoint; (ii) inhibition of the DDR pathway; (iii) inhibition of secretory pathways; and (iv) enhancement of depletion of senescent cells.

The first approach encompasses facilitation of movement through the G2/M phase by overcoming the G2/M checkpoint. This can be done at three different levels: (i) using p53 inhibitors [34,79]; (ii) using histone deacetylase inhibitors [73]; and (iii) performing contralateral nephrectomy of the undamaged kidney after unilateral IRI [96,97]. The use of p53 inhibitors has already been successful in AAN [79] and cisplatin-induced nephropathy [78]. Experiments showed that inhibition of p53, with pifithrin-α or with the use of p53-deficient mice, leads to a reduction of renal lesions. Nevertheless, it is important to note that almost 50% of human cancers involve p53 deletions or mutations. Additionally, in mice, p53 deficiency is associated with a high frequency of spontaneous cancers [98,99]. Therefore, interventions should be undertaken with great caution and for a limited time period. It has been reported that short-term inhibition of p53 with subsequent restauration of the renal function may be a safe approach [100,101].

At the second level, histone deacetylase inhibition has also proven its efficiency in IRI mice and toxin-induced AKI in zebrafish. Inhibition with 4-(phenylthiol)butanoic acid (PTBA) accelerates recovery, increases proliferation and reduces G2/M arrest in surviving PTCs [73]. Finally, the third level involves contralateral nephrectomy. More than 60 years ago, Hinman for the first time described the theory of renal counterbalance. This theory was based on the well-known observations that unilateral kidney injury was followed by compensatory changes in the opposite unharmed kidney [102,103]. Animal experiments have shown that this hypertrophic compensatory reaction of the normal kidney suppresses recovery of the injured kidney which eventually leads to atrophy of the damaged kidney. However, Hinman found that contralateral nephrectomy of the undamaged kidney induced a remarkable recovery of the damaged kidney. This contralateral nephrectomy also leads to a reduction in G2/M-arrested cells [34]. The exact mechanism that drives this cell cycle effect remains unclear and deserves further research.

The second therapeutic approach involves inhibition of the DDR pathway, as G2/M arrest mainly occurs through the activation of CHKs downstream of the ATM/ATR pathway. Incubation of HK-2 cells with KU-55933, an ATM inhibitor [104], reduced the fraction of cells in G2/M arrest by almost 50% [34]. It should be noted that intervention with DDR is tricky as this pathway also stimulates DNA repair and controlled cell death [55].

Third, JNK is known to enhance *TGFβ* and *CCN2* gene expression and fibrosis during G2/M arrest. Therefore, inhibition of JNK activity could protect the kidney against fibrosis [34]. An important side note is that this treatment does not directly decrease the number of G2/M-arrested cells, but rather affects the accompanying pro-fibrotic effect. As mentioned before, secreted pro-fibrotic cytokines like IL-8 lead to the activation of NF-κβ and the p83-MAPK pathways which are both responsible for cell senescence [82].

Finally, the last approach involves enhancement of depletion of senescent cells as these cells stimulate maladaptive repair by the factors they secrete [105]. With this approach it is important to selectively deplete such cells as otherwise it could potentially result in loss of cells which under physiological conditions require no or only slow-rate cell divisions. In this context, it is worth mentioning that Baker et al. demonstrated the principle of removing senescent cells, expressing high levels of p16 and p21, by administration of a homodimerizer drug to transgenic animals [18,106]. Life-long removal of senescent cells delayed tissue dysfunction in adipose tissue, eye and skeletal muscle [18,106,107].

## 5. Conclusions

In this review, we focussed on cell cycle behaviour of PTCs in the injured kidney by providing a molecular overview per cell cycle phase. It is clear that renal injury and repair as well as progression to chronic kidney disease are intimately connected via cell cycle events that often lead to cell cycle arrest. Dividing cells that hit a phase too soon or stay in a phase too long become maladaptive and frequently lead to development of CKD [108]. Development of therapeutic strategies will require profound molecular insight in the complete set of cell cycle associated pathways such that delicate interventions without (severe or even life-threatening) side effects can be developed. Although solid insights have already been obtained, a recent in vitro study revealed that the road is still long as it identified over 14,000 phosphorylation events related to more than 3600 proteins for one round of the cell cycle [109]. This unprecedented illustration of the complexity of cellular proliferation will undoubtedly nourish future cell cycle research in the field of AKI and CKD.

## Abbreviations

AAN	Aristolochic acid toxic nephropathy
AKI	Acute kidney injury
APC	Anaphase-promoting complex
ATM	Ataxia telangiectasia mutated protein
ATR	Ataxia telangiectasia and Rad3-related protein
CCN2	Connective tissue growth factor 2
cdc25	Cell division cycle 25
CDK	Cyclin-dependent kinase
CHK	Checkpoint kinase
CIP	CDK2 interacting protein
CKD	Chronic kidney disease
CKI	Cyclin-dependent kinase inhibitor
Col I	Collagen 1
CTGF	Connective tissue growth factor 2
CVD	Cardiovascular disease
CXCR2	C-X-C motif chemokine receptor
DDR	DNA-damage response
E2F	E2 transcription factor
ECM	Extracellular matrix
EGFR	Epidermal growth factor receptor
FAN1	Fanconi anemia-associated nuclease 1
FDA	Food and drug administration
GFR	Glomerular filtration rate
IL-8	Interleukin-8
INK4	Inhibitors of CDK4
IRI	Ischemia-reperfusion injury
JNK	c-Jun NH2-terminal kinase
KDIGO	Kidney disease: Improving global outcomes
KIP	Kinase inhibiting protein
MAPK	Mitogen-activated protein kinase
NADPH	Nicotinamide adenine dinucleotide phosphate
NF-κβ	Nuclear factor kappa beta
PTBA	4-phenylthiol-butanoic acid
PTC	Proximal tubular epithelium cell
Rb	Retinoblastoma protein
ROS	Reactive oxygen species
SASP	Senescent associated secretory phenotype
TGFβ	Transforming growth factor beta
UUO	Unilateral ureteric obstruction
